# Analytic Gradients for Selected Configuration Interaction

**DOI:** 10.1021/acs.jctc.2c01062

**Published:** 2023-01-19

**Authors:** Jeremy P. Coe

**Affiliations:** Institute of Chemical Sciences, School of Engineering and Physical Sciences, Heriot-Watt University, Edinburgh, EH14 4AS, United Kingdom

## Abstract

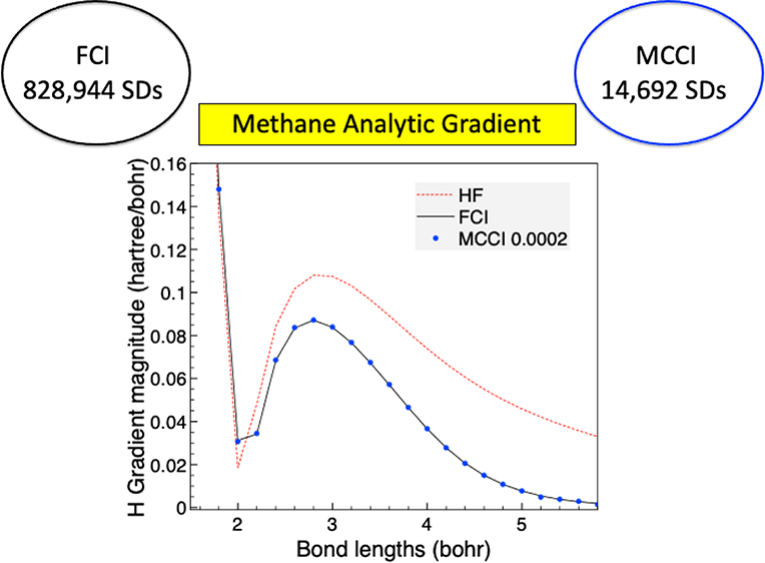

We develop analytic gradients for selected configuration interaction wave functions. Despite all pairs of molecular orbitals now potentially having to be considered for the coupled perturbed Hartree–Fock equations, we show that degenerate orbital pairs belonging to different irreducible representations in the largest abelian subgroup do not need to be included and instabilities due to degeneracies are avoided. We introduce seminumerical gradients and use them to validate the analytic approach even when near degeneracies are present due to high-symmetry geometries being slightly distorted to break symmetry. The method is applied to carbon monoxide, ammonia, square planar H_4_, hexagonal planar H_6_, and methane for a range of bond lengths where we demonstrate that analytic gradients for selected configuration interaction can approach the quality of full configuration interaction yet only use a very small fraction of its determinants.

## 1 Introduction

Selected configuration interaction (CI) can enable accurate electronic structure calculations using only a very small fraction of the determinants needed for full configuration interaction (FCI). This permits larger systems to be tackled without requiring an active space to be chosen, which often needs expert knowledge, and without the resulting risk of bias. Selected CI is particularly important for situations where efficient approaches based on small corrections to a single determinant struggle. These are often referred to as multireference problems, which can include excited states, stretched bonds, and molecules containing transition metals. There is a resurgence of interest in developing and applying this concept^1−17^ beyond the original perturbative approach to selection envisaged by Huron, Malrieu, and Rancurel.^18^ However, to fully realize selected configuration interaction’s potential and enable its accurate and efficient use for geometry optimization or dynamics, then it is crucial to develop analytic gradients for the method.

To improve efficiency and accuracy, analytic gradient methods use the integrals of the derivatives of atomic orbitals evaluated analytically rather than the numerical derivative of the energy. For general Hartree–Fock wave functions and second-order Møller–Plesset perturbation theory (MP2), this was developed by Pople, Krishnan, Schlegel, and Binkley.^19^ Analytic gradients were created for the complete active space self-consistent field (CASSCF) approach in ref (20). and for standard configuration interaction in ref (21). Coupled cluster analytic derivatives have been created for CCSD in ref (22), CCSD(T) in ref (23), coupled cluster using Bruekner doubles in ref (24), and recently for CCSD(T) with the density fitting approximation.^25^ Analytic gradients have been developed for multireference CI (MRCI),^26,27^ state-averaged CASSCF,^28^ and state-specific^29^ then multistate^30,31^ internally contracted CASPT2. See ref (32) for a review of gradients for these multireference methods. Analytic gradients have also been created for the state-specific density matrix renormalization group (DMRG) approach,^33,34^ and for full configuration interaction quantum Monte Carlo (FCIQMC)^35^ for which the analytic gradients have very recently been further developed to allow frozen core orbitals and active spaces.^36^ Impressive recent work has also created analytic gradients for CASSCF-like approaches where selected CI is used instead of CASCI, and the orbitals are optimized. These have been developed for adaptive sampling CI SCF (ASCI-SCF),^37^ ASCI-SCF with second-order perturbation theory,^38^ and the heat bath configuration interaction self-consistent field method (HCISCF).^39^ Explicitly correlated (F12) methods MP2-F12 and CCSD(T)-F12 have recently had analytic gradient technology developed,^40,41^ as has state-averaged DMRG using an approximate parametrization of the matrix product state wave function to avoid the computationally challenging transformation to the configuration basis.^42^

Unlike standard truncated configuration interaction, for example limiting to single and double substitutions (CISD) where the energy is unaffected by rotations between pairs of occupied Hartree–Fock molecular orbitals (MOs) or between pairs of unoccupied MOs,^21,43^ the challenge for selected CI analytic gradients is that the energy is not necessarily invariant to rotations between *any* pair of MOs. This means that to include the contribution from the change in the MO coefficients to the gradient, the coupled perturbed Hartree–Fock (CPHF) equations^44^ have to be considered, in principle, for all pairs of MOs. These equations have factors of (ϵ_*p*_–ϵ_*q*_)^−1^ for orbitals *p* and *q* that are either both occupied or both unoccupied in the Hartree–Fock determinant where ϵ_*p*_ is the energy of orbital *p*. Hence ostensibly degeneracies or near degeneracies will lead to difficulties. However, we demonstrate in this paper that pairs of orbitals belonging to different irreducible representations (irreps) do not contribute. We then find that, for the range of molecules considered, degeneracies due to symmetry split into different irreps when the largest abelian subgroup is used and therefore do not pose a problem. In addition, we show that near-degeneracies do not cause difficulties for selected CI analytic gradients when a geometry without symmetry is as close as 10^–3^ Å to a high-symmetry structure, or when a larger basis set is used.

The general approach to selected CI is to iteratively build up a compact wave function by iteratively adding/removing configurations based on some criteria and diagonalizing the Hamiltonian matrix. This is repeated until a convergence is reached. In this work we use the approach of Monte Carlo configuration interaction (MCCI)^45−47^ where configurations are added randomly but removed if the magnitude of their coefficient in the resulting wave function is less than a cutoff. This form of selected CI has most recently been used to calculate X-ray scattering results that can approach FCI for multireference problems despite using a very small fraction of the number of determinants.^48^ Although we use MCCI as an example, we emphasize that the analytic gradient development in this work is applicable to any selected CI method.

In this paper, we first discuss fully numerical derivatives and how they can have accuracy problems from needing a step size and require at least one extra selected CI calculation for each of the *O*(3*N*_*A*_) coordinates where *N*_*A*_ is the number of atoms. Hence they will easily become computationally costly for larger molecules. We then introduce seminumerical selected CI derivatives where only multiple Hartree–Fock computations are needed. However, this still depends on a step size and the atomic orbital (AO) integrals will have to be transformed to MO integrals each time giving a cost scaling for the derivatives of *O*(3*N*_*A*_*M*^5^) where *M* is the number of basis functions. The theory of CI analytic gradients using RHF MOs is then briefly presented where the Z vector approach^49^ means that the CPHF equations only have to solved once and the 3*N*_*A*_ factor is removed from the scaling of the integral transformations. The implementation of this for selected CI using the AO integrals of Libcint^50^ accessed via PySCF^51,52^ is then discussed. Next we demonstrate that degenerate pairs of MOs belonging to different irreps do not contribute to the selected CI analytic gradient.

We test two things in this work: the analytic gradient approach for selected CI by comparing it with semi numerical gradients, and the accuracy of using compact selected CI wave functions for analytic derivatives by comparing them with FCI analytic gradients. We use the method on carbon monoxide, which has doubly degenerate MOs, and find that the seminumerical derivatives can approach the analytic result as the step size is lowered even when the basis size is increased. It is then shown that the graph of the FCI gradient with respect to bond length can be essentially reproduced by using analytic selected CI gradients applied to MCCI with a cutoff of 2 × 10^–4^. Similar results are seen for ground-state and excited ammonia, where now we can also test the method on a structure without symmetry which is as near as 10^–3^ Å to the trigonal planar geometry. In this case, the analytic derivatives did not have problems, but the step size needed to be smaller for the seminumerical approach. We also consider square planar H_4_ where the analytic selected CI gradients are demonstrated to work well for a broken symmetry geometry, and essentially FCI quality gradients could be calculated from MCCI as the bond lengths were varied except for one point where MCCI had difficulties converging on a multireference wave function. Although hexagonal planar H_6_ has multiple doubly degenerate MOs, we show that the approach for selected CI gradients can work well even with a slightly symmetry broken geometry, and with the use of a ghost atom to break symmetry while maintaining the symmetric geometry. As the bond lengths of the hexagonal planar structure were varied, MCCI gradients were seen to give essentially FCI quality results. Finally we look at methane where again MCCI could give practically FCI quality gradients using a very small fraction of the full configuration space. Despite triply degenerate MOs in this case, the analytic selected CI gradients did not have problems but the seminumerical method required a very small step size at short bond lengths if the tetrahedral geometry was slightly modified to break symmetry.

## 2 Theory

The most straightforward approach to the derivative of the electronic energy with respect to nuclear coordinates (e.g., *X*_*A*_) is fully numerical using finite differences
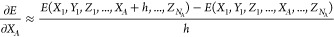
1

where *N*_*A*_ is the number of atoms. For Cartesian coordinates, this requires 3*N*_*A*_ computationally expensive selected CI calculations that can be reduced to 3*N*_*A*_ – 6 for a nonlinear molecule if internal coordinates are used, but this still scales as *O*(3*N*_*A*_) . For highly symmetric systems then symmetry can be used to reduce the number of degrees of freedom. However, for geometry optimization not restricted to a symmetry, or for dynamics in general, then all coordinates would need to be considered. This forward difference approach has error of *O*(*h*) that can be improved to *O*(*h*^2^) by using central differences but at a cost of double the number of selected CI calculations. Unfortunately one cannot just keep lowering *h* as if it goes below the precision for the computation then accuracy will begin to decrease again. In addition, the stochastic nature of some approaches to selected CI can exacerbate the error of fully numerical gradients.

### 2.1 Seminumerical Derivatives

The selected CI wave function is a sum of Slater determinants |Ψ⟩ = ∑_*i*_*c*_*i*_ |Φ_*i*_⟩ so the change in energy in principle depends on how the coefficients *c*_*i*_ vary with a nuclear displacement. However, this contribution is  where the first term is zero as the *c*_*i*_ are variationally determined. The electronic energy may be written in terms of spatial molecular orbitals as
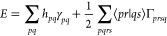
2Here the one-electron integrals are
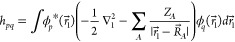
3where *R⃗*_*A*_ is the position of nucleus *A* and *Z*_*A*_ is its charge, while *r⃗*_1_ is the electron coordinate and the two-electron integrals are
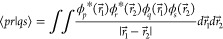
4

The one and two-particle reduced density matrices (γ_*pq*_ and Γ_*prsq*_) only depend on the *c*_*i*_ and the occupation of the Slater determinants (see, e.g. ref (53)); hence, they will not be affected by the infinitesimal change in geometry so the gradient can be written as
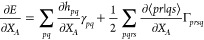
5

This can be used for what we term a seminumerical approach where only the derivatives of the integrals need to be approximately calculated numerically using forward differences. This is achieved using the molecular orbital (MO) integrals resulting from multiple Hartree–Fock calculations at different geometries. The benefit of this is that only one selected CI calculation needs to be run and selection methods that have an element of randomness will not cause problems. The relative simplicity is also attractive, and there will not be possible problems from (near) degenerate MOs. Once the two-particle density matrix (2RDM) is calculated using the efficient approach of ref (53), then multiplying this by the integral derivatives scales as *O*(*M*^4^) where *M* is the number of MOs. However, the integrals have to be calculated then transformed from AOs to MOs, and using the four-index transformation,^54^ this scales as *O*(*M*^5^) . This has to be done for each coordinate so the overall scaling for the derivatives will be *O*(3*N*_*A*_*M*^5^) . Furthermore, there will still be a dependence on the step size *h*. To improve accuracy and remove the requirement to run *O*(3*N*_*A*_) integral transformations, we next discuss the more complicated fully analytic derivatives.

### 2.2 Analytic Derivatives

We briefly sketch the derivation of analytic CI derivatives using RHF MOs which we partly base on ref (21) and ref (55). As a change in the geometry will cause the MO coefficients (*C*_*i*_) and the AOs (χ_μ_) to vary then from ref (24), the MOs will become
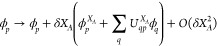
6Here the *U*_*qp*_^*X*_*A*_^ give the change in MO coefficients due to the perturbation and are found by solving the coupled-perturbed Hartree–Fock (CPHF) equations of Gerratt and Mills,^44^ while ϕ_*p*_^*X*_*A*_^ means that the derivatives of AOs transformed to the MO basis are used, i.e., . For CI gradients, this leads to (see e.g. ref (21))
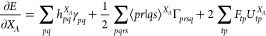
7where
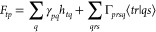
8is often termed the CI Lagrangian. It may appear that eq 7 requires the analytic derivative of the two-electron integrals to be transformed to the MO basis for all 3*N*_*A*_ coordinates, but the 2RDM can be back transformed just once instead,^21^ where we have used greek letters for AOs:
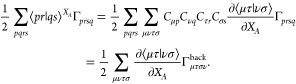
9Here Γ_*μ τ σ ν*_^back^ = ∑_*pqrs*_*C*_*μ p*_*C*_*ν q*_*C*_*τ r*_*C*_*σ s*_ Γ_*prsq*_ and a corresponding procedure is also used for the one-electron integrals.

*F*_*pq*_ is the same as the generalized Fock matrix in the creation of CASSCF^56^ where *ΔF*_*pq*_ = *F*_*pq*_ – F_*qp*_ is the change in energy due to a small rotation between orbitals *p* and *q*. Hence if the energy is invariant with respect to rotating these orbitals then *ΔF*_*pq*_ = 0 and this is exploited by using *ΔF*_*pq*_ in eq 7. The standard approach to achieve this uses the orthonormality of the MOs ⟨ϕ_*p*_|ϕ_*q*_⟩= *S*_*pq*_ = δ_*pq*_ so . Then using eq 6 this can be written as *U*_*qp*_^*X*_*A*_^ + *U*_*pq*_^*X*_*A*_^ + *S*_*pq*_^*X*_*A*_^ = 0. This enables the last term of eq 7 to be rearranged to 2∑_*t*>*p*_*ΔF*_*tp*_*U*_*tp*_^*X*_*A*_^ – ∑_*tp*_*F̃*_*tp*_*S*_*tp*_^*X*_*A*_^ where
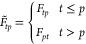
10Combining these equations gives the following for the analytic gradient of the electronic energy
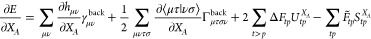
11

For FCI with no frozen orbitals then the energy is invariant to orbital rotations, so from ref (56) all *ΔF*_*tp*_ are zero and the *U*_*tp*_^*X*_*A*_^ for the change in MO coefficients do not need to be calculated. Otherwise the coupled perturbed Hartree–Fock (CPHF) equations^44^ need to be solved where the perturbation is a nuclear displacement. This uses the MO basis to lead^44^ to a set of linear equations which, in the notation of this paper, are
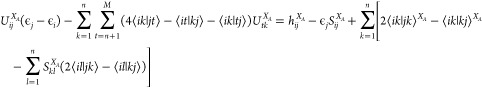
12where *j* ranges over the *n* = *N*_*e*_/2 double occupied RHF MOs, *i* over the *M*–*n* unoccupied MOs in RHF and ϵ_*i*_ are the Hartree–Fock orbital energies. We solve these equations by mapping to one variable using *i*, *j* →*s* where *s* = *j* + *n*(*i*–*n*–1) so they are in the standard form of *GU⃗*^*X*_*A*_^ = *y⃗* for a system of linear equations. For CISD analytic gradients, with no frozen orbitals, this would be sufficient as rotations between RHF occupied MOs or between unoccupied MOs do not change the energy so *ΔF*_*tp*_ = 0 for these pairs. Hence, only pairs where one is occupied in RHF and the other unoccupied need to be considered. However, for selected CI this is not generally the case so other *U*_*pq*_^*X*_*A*_^ values need to be calculated where both *p* and *q* are occupied RHF orbitals or both are unoccupied. This can be done^44^ using the known *U*_*ij*_^*X*_*A*_^ in a rearranged eq 12
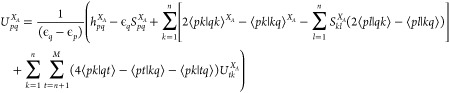
13

The  factor suggests there may be issues in selected CI analytic gradients if orbital energies are degenerate or near degenerate. However, we will demonstrate later in this work that this is not such a problem as it may first appear.

There is a scaling challenge with this approach to CPHF as eq 12 needs to be solved for each coordinate. There are *n*(*M*–*n*) equations in the linear system, so solving this using Gaussian elimination would be expected to scale as *O*(*M*^3^) as long as the number of basis functions is much greater than the number of electrons. However, each time the two-electron derivatives have to be transformed to the MO basis at a cost of *O*(*M*^5^) so to avoid an overall *O*(3*N*_*A*_*M*^5^) scaling, we use the Z-vector approach of Handy and Schaefer.^49^ For *j* an occupied RHF MO and *i* an unoccupied RHF MO then eq 12 can be written as
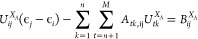
14When mapping *i* and *j* to one variable this can be put in the form *G U⃗*^*X*_*A*_^ = *B⃗*^*X*_*A*_^ where *G* is free of derivatives. The 2∑_*j*>i_*ΔF*_*ji*_*U*_*ji*_^*X*_*A*_^ contribution to the analytic energy gradient (eq 11) is then
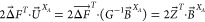
15where *Z⃗*^*T*^*G* = *ΔF⃗*^*T*^ so *G*^*T*^*Z⃗* = *ΔF⃗* which only needs to be solved once rather than for each coordinate. To include the contribution from other pairs of MOs the 2∑_*t*>p_*ΔF*_*tp*_*U*_*tp*_^*X*_*A*_^ term in eq 11 can be written using eq 13 where *i* is an unoccupied RHF MO, *j* is an occupied RHF MO, and *p*, *q* are any other pair of orbitals, i.e., both RHF occupied or both RHF unoccupied:
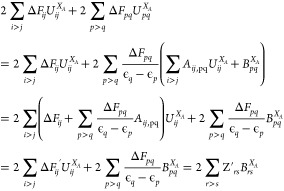
16Here *r*, *s* range over all MOs while for *i*, *j*, we have  and *G*^*T*^*Z⃗*′ = *ΔF⃗*′ is solved, then for *p*, *q*.

However, 2∑_*r*>*s*_*Z*′_*rs*_*B*_*rs*_^*X*_*A*_^ would still have *O*(3*N*_*A*_*M*^5^) scaling if the two-electron derivatives were transformed to the MO basis each time but we leave them in the AO basis and back transform *Z*′. The two-electron derivatives in *B*_*rs*_^*X*_*A*_^ are ∑_*k* = 1_^*n*^ 2 ⟨*rk*|*sk*⟩^*X*_*A*_^–⟨*rk*|*ks*⟩^*X*_*A*_^ so the contribution to 2∑_*r*>s_*Z*_*rs*_^′^*B*_*rs*_^*X*_*A*_^ is
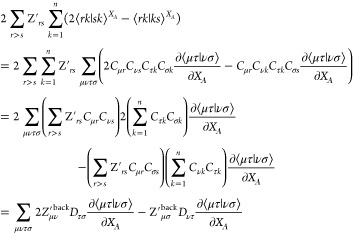
17where *Z*_*μ ν*_^′back^ = ∑_*r*>s_*Z*_*rs*_^′^*C*_*μ r*_*C*_*ν s*_ and *D*_τ_ σ is the RHF density matrix *D*_τ_ σ = 2∑_*k* = 1_^*n*^*C*_*τ k*_*C*_*σ k*_. Hence, we have removed the *O*(*M*^5^) scaling for each coordinate and the calculation cost is now *O*(3*N*_*A*_*M*^4^).

The analytic derivative of the total selected CI energy then includes the contribution from the nuclear–nuclear repulsion and is written as
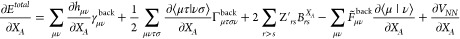
18where we have also back transformed *F̃*, greek letters are for AOs, *r*, *s* range over all MOs for which *ΔF*_*rs*_ ≠ 0 and eq 17 is used in the computation of 2∑_*r*>s_*Z*′_*rs*_*B*_*rs*_^*X*_*A*_^. The nuclear–nuclear repulsion , where *I*, *J* range over all atoms, has derivative .

### 2.3 Implementation

The AO integrals are calculated using the Libcint library of Sun^50^ which is interfaced through PySCF.^51,52^ We also use PySCF to provide the RHF MO coefficients and energies. The integrals of the derivatives of Gaussian AOs are output with respect to the electronic coordinates, e.g.,  for the overlap, which we assemble into nuclear coordinate, e.g., *X*_*A*_, derivatives by changing the sign and only including those where μ is on atom *A*. The one-electron operators also contain a dependence on *X*_*A*_ through  where *i* ranges over the electrons and *J* the nuclei. Here *R*_*iJ*_ = |*r⃗*_*i*_–*R⃗*_*J*_| and the derivative of the operator is
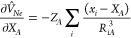
19

The integrals via PySCF are in the form  where the derivatives are with respect to electronic coordinates. We have that  From integration by parts we also have  hence . As  then we can construct the contribution of the derivative of the one-electron operators to the gradient from the PySCF output. We use the MCCI program^47^ to calculate the selected CI wave functions and the approach from ref (53) to efficiently compute the 2RDMs.

### 2.4 Degeneracies

Due to the  term in the analytic gradient, where *p* and *q* are either both occupied RHF orbitals or both unoccupied, then degenerate orbitals could cause difficulties as, unlike CISD, *ΔF*_*pq*_ is not necessarily zero in this case. However, we show that as long as a degenerate pair *t*, *p* belong to different irreps of the largest abelian subgroup of the molecule then *F*_*tp*_ = 0.

If we have that orbital *t* is of irrep *I*_*t*_ and orbital *p* is of irrep *I*_*p*_ where *I*_*t*_ ≠ *I*_*p*_, then for


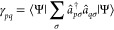
20

to be nonzero, *p* and *q* must have the same irrep otherwise ∑_σ_*â*_*pσ*_^†^*â*_*qσ*_ |Ψ⟩ will have a different irrep to ⟨Ψ|. Hence *I*_*p*_ = *I*_*q*_ for the first sum in the expression for *F*_*tp*_ (eq 8). However, then *I*_*t*_ ≠ *I*_*q*_ and the *h*_*tq*_ integrals are all zero.

For the second term, the 2-RDM
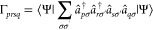
21

requires *I*_*s*_ × *I*_*q*_ = *I*_*p*_ × *I*_*r*_ for it to be nonzero while the two-electron integrals ⟨*tr*|*qs*⟩ need *I*_*t*_ × *I*_*r*_ = *I*_*q*_ × *I*_*s*_. For both to be nonzero then *I*_*p*_ × *I*_*r*_ = *I*_*t*_ × *I*_*r*_ but then *I*_*t*_ = *I*_*p*_, yet we have *I*_*t*_ ≠ *I*_*p*_, so the second term must also be zero. To allow for numerical errors, we set |*ΔF*_*tp*_|values less than 10^–8^ to zero.

Degeneracies due to symmetry will belong to an irrep with dimension greater than one in the full point group of the molecule, but we expect these orbitals to split into different irreps when the largest abelian subgroup is used, and this is indeed what we demonstrate for a range of molecules in the Results.

For geometries that are without symmetry yet are very close to a high symmetry structure, then near degeneracies might cause issues. Here approaches similar to those to deal with intruder states in multireference perturbation theory (see e.g. ref (57)) could be employed if there are problems. However, for the systems in this work, we find below that we can get to within 10^–3^ Å; of a high symmetry geometry without difficulties.

## 3 Results

### 3.1 Carbon Monoxide

We first consider CO using the 6-31G basis with two frozen orbitals. We use the *C*_2*v*_ point group and look at the ground *A*_1_ state with *M*_*s*_ = (1/2)(*N*_α_–N_β_) = 0. The full *C*_∞*v*_ point group has irreps of dimension 2, so there can be doubly degenerate orbital energies by symmetry. We find that these degenerate pairs occur but all split into different irreps when using this point group’s largest abelian subgroup of *C*_2*v*_.

**Figure 1:**
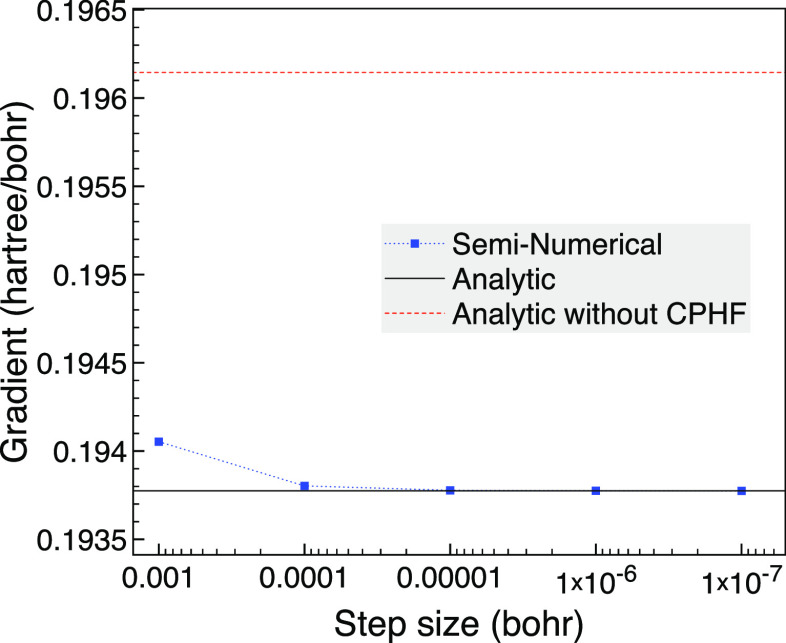
Analytic and seminumerical gradients for CO with a bond length of 3 bohr using the 6-31G basis set, two frozen orbitals and MCCI with a cutoff of 5 × 10^–4^.

We verify the selected CI analytic gradients for this system by comparing them with the seminumerical approach with decreasing step size when using MCCI with a cutoff of 5 × 10^–4^. When *ΔF* is nonzero, the smallest orbital energy difference for this example is around 6 × 10^–2^ hartree. Figure 1 shows that for a bond length of 3 bohr, the seminumerical derivatives tend to the analytic result as the step size is lowered with negligible difference on the scale of the graph when a step size of *h* = 10^–6^ bohr is reached. We also plot the analytic derivative without the CPHF term as an approximation that assumes *ΔF*_*rs*_ = 0 for all pairs of orbitals and so removes the possibility of problems from (near) degenerate orbitals. The difference from not including the CPHF contribution is noticeable in the plot but is only around 1.2% of the gradient.

**Figure 2:**
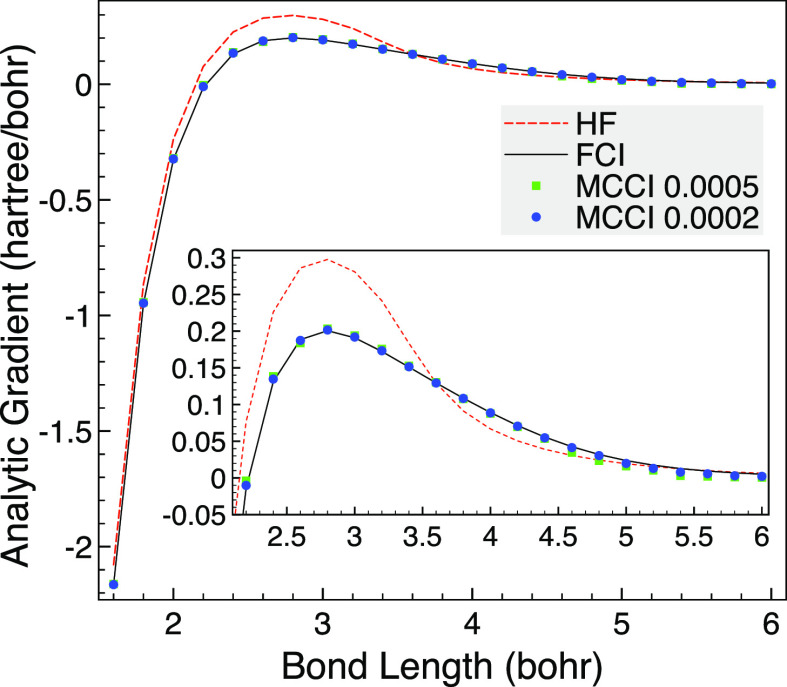
HF, FCI, and MCCI analytic gradients for CO as the bond length is varied using the 6-31G basis set, two frozen orbitals and the C_2v_ point group. Inset: Enlarged view of the gradient curve.

The MCCI analytic gradients are then compared with the HF and FCI results as the bond length is varied. To test our implementation using Libcint^50^ integrals via PySCF,^51,52^ we use a different program for this: the analytic FCI gradients are calculated using a full space MCSCF calculation with two frozen orbitals in MOLPRO.^58^ We see in Figure 2 that the HF gradient is noticeably different from FCI until the bond length is very long. In contrast, MCCI with a cutoff of 5 × 10^–4^ is very close to FCI although there is small difference at very stretched bonds, but then the gradient is close to zero anyway. This may be due to the use of Slater determinants allowing a different spin state to be arrived at as dissociation is approached, which could be avoided by using configuration state functions (CSFs) when going beyond these proof of concept results. The MCCI results are improved upon by lowering the cutoff to 2 × 10^–4^ where now the MCCI gradient is essentially indistinguishable on the scale of the graph.

We quantify the accuracy using the root-mean-square error (RMSE) over all *N*_*g*_ geometries, *N*_*A*_ atoms and the coordinates for each atom. Although the gradient can be described with respect to a single coordinate for a diatomic, we consider all 3*N*_*A*_ = 6 values, despite 4 being zero, for consistency with the larger systems investigated later in this work.
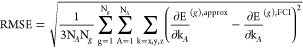
22We see in Table 1 that the error is over 20 times smaller with the lowest MCCI cutoff than with HF and the mean number of Slater determinants (SDs) used for this is a small fraction of the FCI space of 4,777,056 determinants.

**Table 1:** Errors Using RMSE When Compared with FCI for the Gradient of CO and Mean Number of Determinants across the 23 Geometries When Employing 6-31G and Two Frozen Orbitals

Method	Gradient Error (hartree/bohr)	Mean Determinants
HF	3.28 × 10^–2^	1
MCCI 1 × 10^–3^	0.525 × 10^–2^	2220
MCCI 5 × 10^–4^	0.330 × 10^–2^	5087
MCCI 2 × 10^–4^	0.147 × 10^–2^	15024

**Figure 3:**
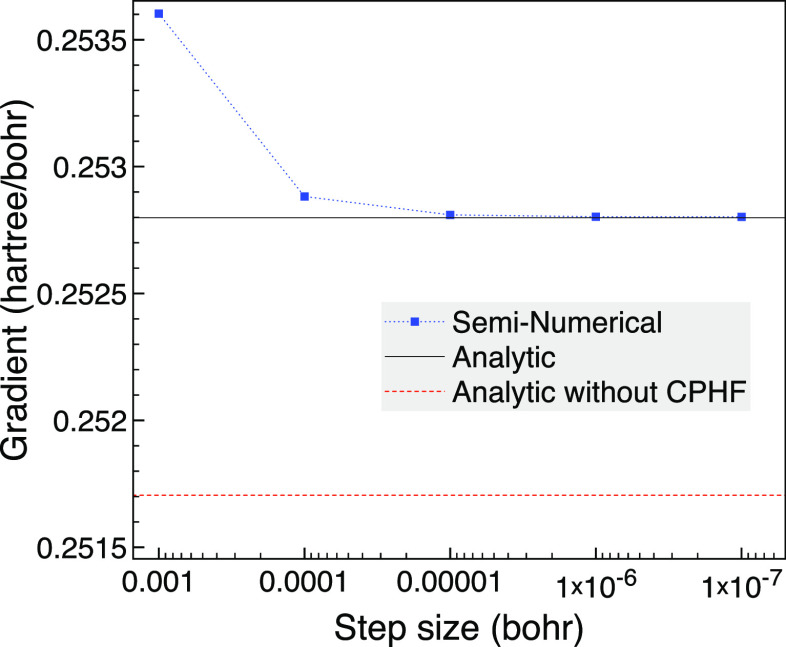
Analytic and seminumerical gradients for CO with a bond length of 3 bohr using the cc-pVTZ basis set, two frozen orbitals, and MCCI with a cutoff of 5 × 10^–4^.

We also test the analytic gradients using MCCI with a cutoff of 5 × 10^–4^ for the bond length of 3 bohr but with a larger basis set of cc-pVTZ that is beyond FCI. The larger basis set should mean that near-degeneracies for the energies of orbitals from the same irrep are more likely. We now find that the smallest energy difference for the orbitals when *ΔF* is nonzero is around 3 × 10^–3^ hartree. Despite this we see in Figure 3 that the seminumerical derivatives again verify the analytic result by approaching it as the step size is lowered. We also check the effect of lowering the cutoff here to 2 × 10^–4^. This causes the number of determinants to increase from 15857 to 54280 and the gradient to slightly lower to 0.241 hartree/bohr. By only considering the derivatives of operators, we test if the Hellmann–Feynman condition is close to being satisfied so that  is a good approximation to the energy gradient. For OC geometry and a cutoff of 5 × 10^–4^, this gives an approximate gradient of −0.027 hartree/bohr for oxygen and 0.071 hartree/bohr for carbon. For the lower cutoff of 2 × 10^–4^ the Hellmann–Feynman theorem results in an approximate gradient of −0.040 hartree/bohr for oxygen and 0.058 hartree/bohr for carbon. Hence, even for this larger basis, the Hellman–Feynman condition is far from being satisfied, and derivatives of orbitals must still be taken into account for the gradient to be accurate.

As there is no route to lower the symmetry of a diatomic by varying the geometry, we next test the approach on ammonia because its structure can be modified to remove symmetries, and we can investigate if this leads to near degeneracies of the same irrep causing difficulties.

### 3.2 Ammonia

We now look at NH_3_ where the larger number of degrees of freedom makes using seminumerical derivatives more cumbersome. Furthermore, the ability of the analytic approach to cope with near degeneracies of the same irrep can be tested by breaking the symmetry of this system.

We start with a trigonal planar geometry and a bond length of 1.8 Å. This geometry has full point group *D*_3*h*_ and therefore degenerate orbital energies if they belong to the irreps of dimension 2. For the 6-31G basis, it indeed has four pairs of doubly degenerate orbital energies but within the *C*_2*v*_ abelian subgroup used the degenerate orbital pairs split into different irreps. The symmetries are then removed by raising the N atom out of the plane by 0.01 Å, while the hydrogen atoms remain in the plane but one bond is lengthened by 0.01 Å and another shortened by 0.01 Å.

**Figure 4:**
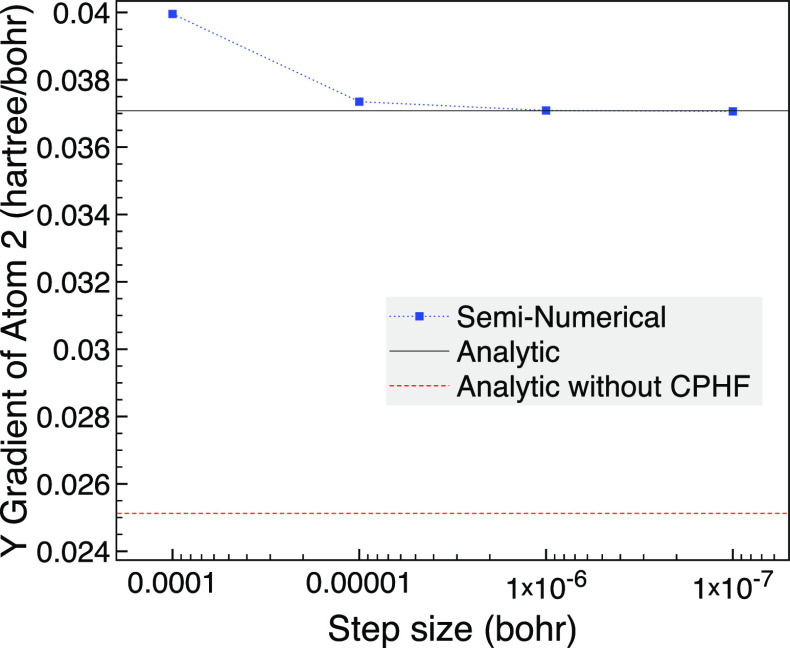
NH_3_ broken symmetry with 0.01 Å changes based on a trigonal planar geometry with 1.8 Å bond lengths, the 6-31G basis set and one frozen orbital using MCCI with a cutoff of 5 × 10^–4^.

For this system, we find that the smallest difference in MO energies is 1.3 × 10^–3^ hartree and |*ΔF*| is not lower than the threshold for any pair of orbitals when using the MCCI wave function. In Figure 4 we consider the *y* gradient of atom 2, which is a hydrogen, and we see that the seminumerical gradient approaches the analytic result as the step size is lowered. Although there are no issues with the analytic gradient method, the seminumerical results using forward differences are more challenging as now a step size of *h* = 0.001 bohr gives a derivative of around 70 hartree/bohr yet this step size was reasonably accurate for the carbon monoxide results.

**Figure 5:**
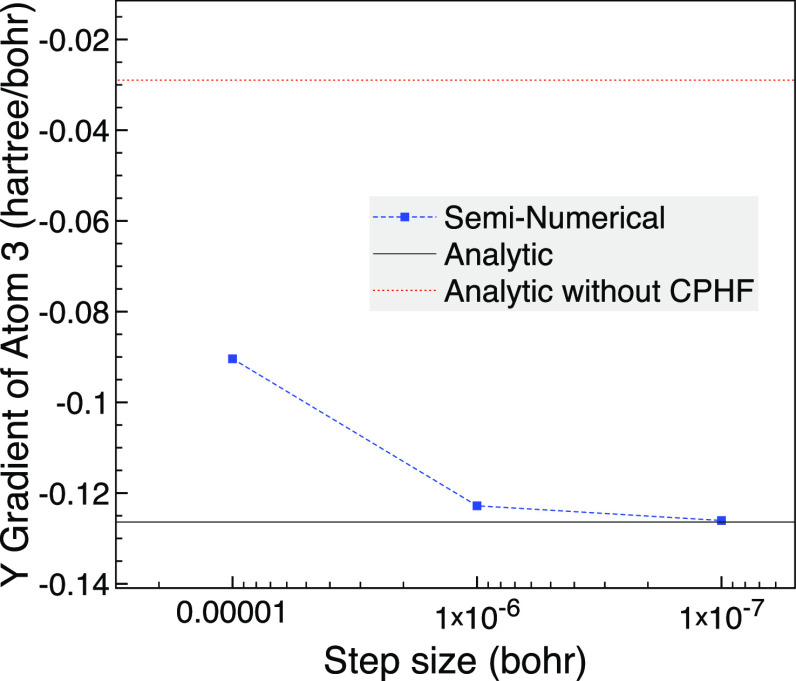
NH_3_ broken symmetry with 10^–3^ Å changes based on a trigonal planar geometry with 1.8 Å bond length, the 6-31G basis set and one frozen orbital using MCCI with a cutoff of 5 × 10^–4^.

We next use the same procedure to break symmetry but with an even smaller change of 10^–3^ Å. Again *ΔF* is non-negligible for all orbital pairs, and now the smallest energy difference between orbitals is 1.3 × 10^–4^ hartree. Figure 5 shows the *y* gradient of atom 3, which is a hydrogen and now has a larger gradient than that of atom 2, and we see that the seminumerical gradient approaches the analytic result. The seminumerical result is even more challenging than the 0.01 Å broken symmetry as now a step size of 10^–4^ bohr is too large and gives 442 hartree/bohr, despite this step size giving a reasonable result for the previous geometry, and we have to go to a 10^–5^ bohr step size for qualitative agreement with the analytic result. The CPHF contribution is very important for the analytic result here as without it the computed gradient would be around 4.4 times smaller. This fits in with all the *ΔF* being non-negligible and small gaps between orbital energies.

**Figure 6:**
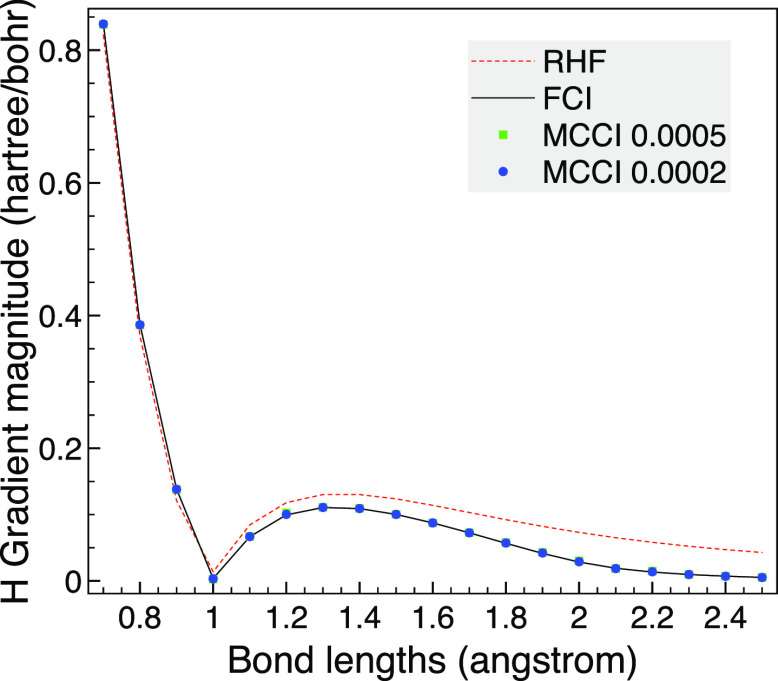
Magnitude of the gradient vector for a hydrogen atom in NH_3_ against bond lengths for the trigonal planar geometry using the 6-31G basis set with one frozen orbital and the C_2v_ point group.

The gradient is now compared with that of FCI using MCSCF in MOLPRO^58^ for the ground state of trigonal planar NH_3_ as the bonds are all lengthened. We see in Figure 6 that the magnitude of the analytic gradient vector for the hydrogens, when calculated using MCCI with a cutoff of 2 × 10^–4^, is practically indistinguishable from FCI and there are only some very small differences in the results with a larger cutoff of 5 × 10^–4^. The HF gradient is noticeably different once the bonds extend past the equilibrium length.

Using the *C*_2*v*_ point group and the 6-31G basis set with one frozen orbital there are 254,561 SDs for the FCI wave function of *A*_1_ irrep, and we see in Table 2 that MCCI uses a small fraction of this. The errors are improved by around an order of magnitude on using MCCI with the largest cutoff considered compared with HF. The smallest MCCI cutoff then reduces the error by almost another order of magnitude.

**Table 2:** Errors Using RMSE When Compared with FCI for the Gradient of Ground-State Trigonal Planar NH_3_ and Mean Number of Determinants across the 19 Bond Lengths Considered When Employing 6-31G and One Frozen Orbital

Method	Gradient Error (hartree/bohr)	Mean Determinants
HF	1.57 × 10^–2^	1
MCCI 1 × 10^–3^	0.0990 × 10^–2^	1649
MCCI 5 × 10^–4^	0.0585 × 10^–2^	3550
MCCI 2 × 10^–4^	0.0248 × 10^–2^	8381

**Figure 7:**
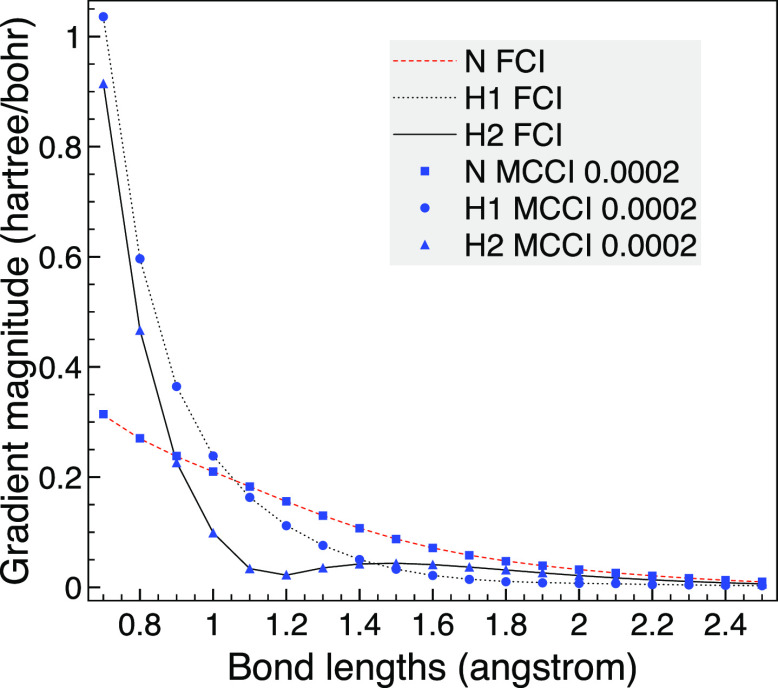
Magnitude of the gradient vectors for three atoms in the first excited *A*_1_ state of NH_3_ against bond lengths for the trigonal planar geometry using the 6-31G basis set with one frozen orbital and the C_2v_ point group.

We now consider the first excited *A*_1_ state for this system which is a *M*_*s*_ = 0 triplet when using Slater determinants. One of the hydrogens now has a different gradient magnitude to the others and the nitrogen gradient is non-negligible. We see in Figure 7 that the analytic gradient magnitudes from MCCI appear to be FCI quality when a cutoff of *c*_min_ = 2 × 10^–4^ is employed.

The errors are portrayed in Table 3 where we see that they are similar in size and behavior to the ground state (Table 2). However, more determinants are needed for the excited states with 12,129 required on average for the lowest cutoff considered.

**Table 3:** Errors Using RMSE When Compared with FCI for the Gradient of the First Excited *M*_*s*_ = 0 *A*_1_ State of Trigonal Planar NH_3_ and Mean Number of Determinants across the 19 Bond Lengths Considered When Employing 6-31G and One Frozen Orbital

Method	Gradient Error (hartree/bohr)	Mean Determinants
MCCI 1 × 10^–3^	0.142 × 10^–2^	2526
MCCI 5 × 10^–4^	0.115 × 10^–2^	5295
MCCI 2 × 10^–4^	0.0262 × 10^–2^	12129

### 3.3 Square Planar H_4_

For H_4_ with a square planar geometry the full point group is *D*_4*h*_ which can have doubly degenerate orbitals by symmetry due to irreps of dimension 2. However, with the cc-pVDZ basis, we do not observe any degeneracies; there are MO energies around 2.5 × 10^–2^ hartree apart, when the bond length is 1.7 bohr, but they belong to different irreps in the *D*_2*h*_ subgroup used. We use geometry (*r*/√2,0,0); (−*r*/√2,0,0); (0,*r*/√2,0); (0,–*r*/√2,0) for a bond length of *r* from orienting by mass in MOLPRO^58^ so that the symmetry group is identified.

Although there are no degeneracies, we check whether slightly breaking the symmetry causes any difficulties due to MOs close in energy not being in different irreps. We distort the *r* = 1.7 bohr geometry with shifts of 0.01 bohr to (*r*/√2,0,0.01); (−*r*/√2,0.01,0); (0,*r*/√2,0); (0,–*r*/√2,0) to do this and now have MO energy differences as small as 6.4 × 10^–4^ in the same irrep that cannot be excluded based on the *ΔF* value from an MCCI 5 × 10^–4^ calculation.

**Figure 8:**
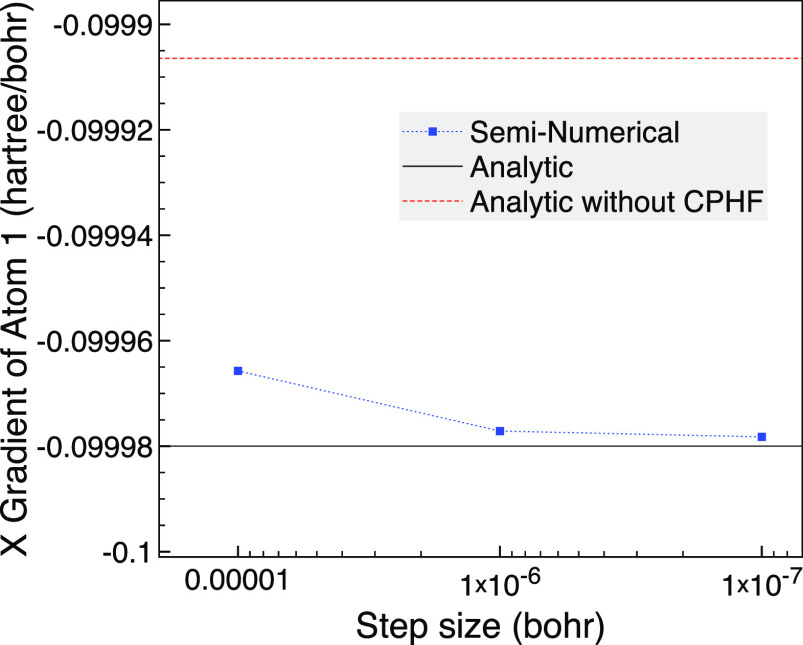
Comparison of seminumerical and analytic X gradient for hydrogen atom one of broken symmetry H_4_ using 10^–2^ bohr changes based on a square planar geometry with 1.7 bohr bond length and the cc-pVDZ basis set using MCCI with a cutoff of 5 × 10^–4^.

We use the seminumerical approach to validate that the small MO energy differences do not prevent an accurate analytic calculation. The seminumerical derivative suggests this system is challenging as for a step size of 10^–4^ bohr the result is very inaccurate with a value of 67, so we only plot from a step size of 10^–5^ bohr. We see in Figure 8 that the seminumerical result approaches the analytic value of the X gradient of atom 1 as the step size is lowered. Although leaving out the CPHF contribution is noticeable on the scale of the graph, the absolute difference is around 7 × 10^–5^. Hence the analytic approach works without problems for a broken symmetry geometry with shifts of 0.01 bohr from the high symmetry geometry.

We now investigate the agreement with FCI for the square planar geometry as all the bonds are stretched. The FCI wave function consists of 5050 determinants for the *A*_*g*_ state when using cc-pVDZ. A larger basis set would have too many orbitals to run the full space MCSCF analytic gradients in MOLPRO^58^ to give the FCI analytic gradients.

**Figure 9:**
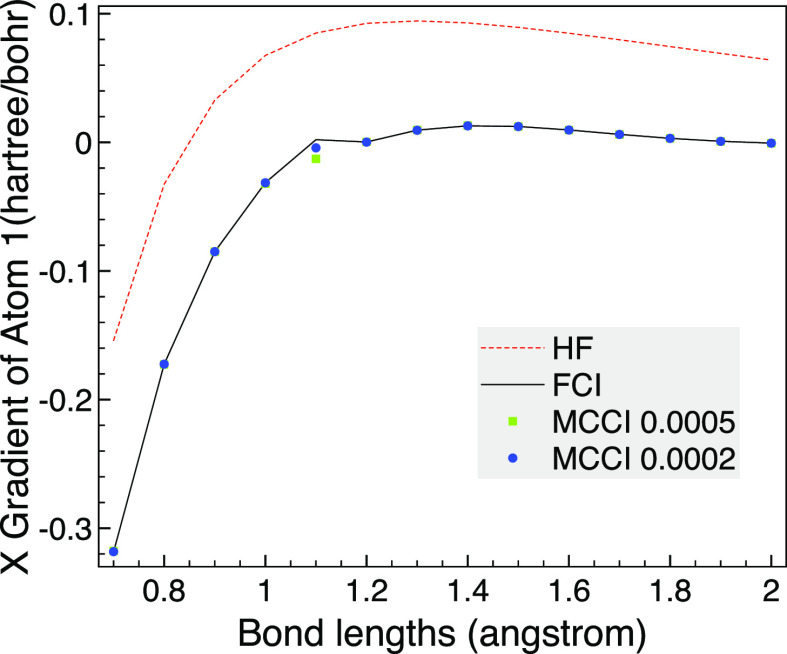
X gradient of atom one in square planar H_4_ against bond lengths using the cc-pVDZ basis set and the *D*_2_*_h_* point group.

Even for the short bond length of 0.7 Å, the MCCI wave function has the largest coefficient of around 0.7 for the 2 × 10^–4^ cutoff, suggesting that this is a multireference problem. Initially the bond length of 1.1 Å gave a very different MCCI gradient to the others, and although the energy was reasonable, its largest wave function coefficient was 0.97 compared to around 0.7 for wave functions at the previous and next geometry. This was rectified by using the 1.0 Å wave function coefficients as a starting point; however, it can be seen in Figure 9 that this result is still slightly different to FCI demonstrating the challenge of this system for MCCI. However, the other MCCI results are essentially FCI quality on the scale of the graph and noticeably better than HF. If we let the MCCI cutoff go to zero, then we recover the FCI gradient for 1.1 Å using our analytic gradient approach.

We see in Table 4 that the gradient errors can be reduced by almost 2 orders of magnitude by using MCCI rather than HF. Interestingly, the gradient errors do not always decrease with lowering the cutoff for this system unlike the previous molecules: unlike the energy, the gradient is not variational and this is a challenging system for MCCI. The difficulty with the 1.1 Å bond length for the 5 × 10^–4^ cutoff will have caused it to have a larger error. Although the most accurate gradient is from the highest cutoff, the lowest cutoff result is similar. We attribute this to the small FCI space and multireference character leading to lower differences between the number of determinants for the different cutoffs. However, even the least accurate MCCI result still reduces the error by over an order of magnitude compared with HF.

**Table 4:** Errors Using RMSE When Compared with FCI for the Gradient of Ground-State Square Planar H_4_ and Mean Number of Determinants across the 14 Bond Lengths Considered When Employing the cc-pVDZ Basis

Method	Gradient Error (hartree/bohr)	Mean Determinants
HF	5.15 × 10^–2^	1
MCCI 1 × 10^–3^	0.0710 × 10^–2^	512
MCCI 5 × 10^–4^	0.227 × 10^–2^	765
MCCI 2 × 10^–4^	0.0974 × 10^–2^	1347

### 3.4 Hexagonal Planar H_6_

H_6_ in a hexagonal planar geometry has *D*_6*h*_ as its full point group so can have doubly degenerate orbitals by symmetry due to irreps of dimension 2. For a bond length of 2.5 bohr and cc-pVDZ, we find 10 pairs of degenerate orbitals, but all pairs split into different irreps when using the largest abelian subgroup of *D*_2*h*_. Hence, we see if by slightly distorting the hexagonal planar geometry to break symmetry, we can induce any difficulties for the selected CI analytic gradients through near degeneracies in the same irrep.

**Figure 10:**
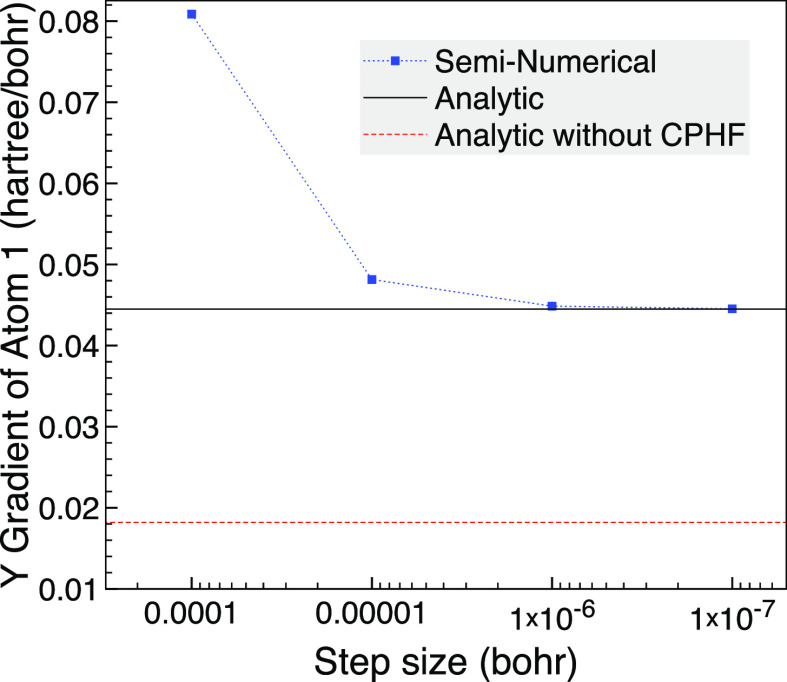
Comparison of seminumerical and analytic Y gradient for hydrogen atom one of broken symmetry H_6_ using 10^–2^ bohr changes based on a hexagonal planar geometry with 2.5 bohr bond lengths and the cc-pVDZ basis set using MCCI with a cutoff of 5 × 10^–4^.

For the hexagonal planar geometry, we have atom *J* coordinates of (*r* cos θ_*J*_, *r* sin θ_*J*_, 0), where *r* is the bond length and θ_*J*_ ranges from θ_1_ = 30° to θ_6_ = 330° in steps of 60°. We break symmetry by shifting atom 2 out of the plane by 0.01 bohr and adding 0.01 bohr to the X coordinate of atom 5. This gives a smallest orbital energy difference of 2.2 × 10^–4^ when *ΔF* is non-negligible. We see in Figure 10 that the analytic derivative result is approached by the seminumerical gradient as the step size is lowered. A larger step size of 0.001 bohr gave a large gradient of 1.3 hartree/bohr so is not plotted. Neglecting the CPHF terms results in a gradient that is less than half the correct value for this system.

**Figure 11:**
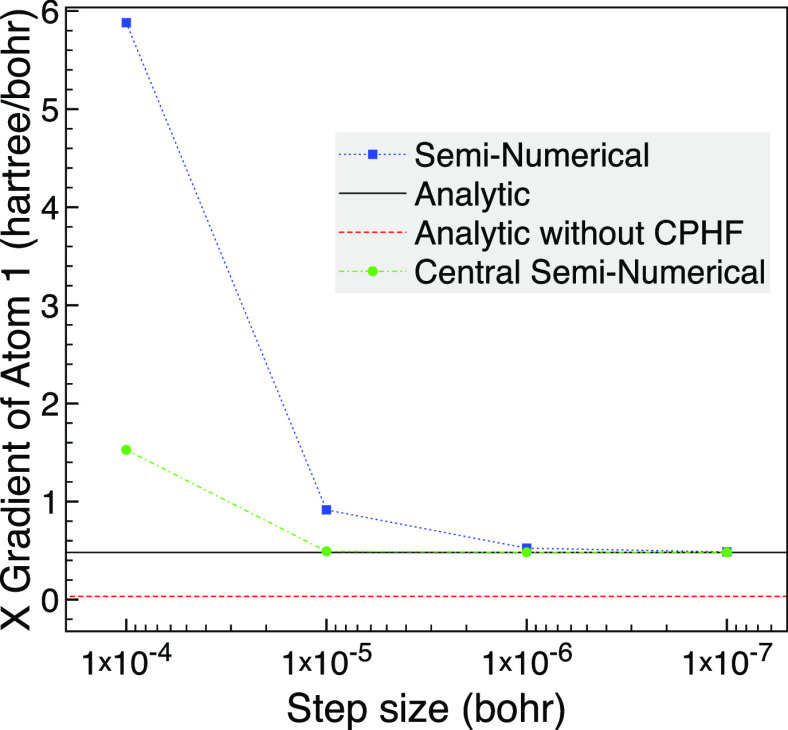
Comparison of seminumerical (forward and central differences) and analytic X gradient for hydrogen atom one of H_6_ in a hexagonal planar geometry with 2.5 bohr bond lengths, a ghost atom at (1,1,1) bohr, the cc-pVDZ basis set using MCCI with a cutoff of 5 × 10^–4^.

We also look at the use of a ghost atom to remove symmetry without changing the hexagonal planar geometry, albeit at the cost of a larger basis size. We place the ghost hydrogen atom at (1,1,1) bohr when using the hexagonal planar geometry with bond lengths of 2.5 bohr. The smallest energy gap between pairs of orbitals that have to be considered is now 3.3 × 10^–5^. We see in Figure 11 that the seminumerical derivative converges to the analytic result as the step size is lowered. We also further verify the results by using central differences for the seminumerical derivative which are more accurate for a given step size but require an additional HF calculation and integral transform for each coordinate of interest. Using central differences, the analytic result is approached faster but a step size of 10^–4^ remains too large for sufficient accuracy.

The use of a ghost atom again demonstrates the accuracy of the selected CI analytic derivatives even when confronted with near degeneracies. However, using a ghost atom does not appear to be a promising strategy to allow high symmetry geometries to be modeled with no symmetry as the MCCI calculation was much more challenging: for a cutoff of 5 × 10^–4^, 4729 determinants were needed compared with 3029 when *D*_2*h*_ symmetry and no ghost atom was used. Despite the larger number of determinants, the MCCI result for the ghost atom system had a slightly higher energy and a larger X gradient on atom 1 than the MCCI value of 0.033 hartree/bohr when not using a ghost atom and exploiting symmetry by using *D*_2*h*_. Without symmetry the FCI space would be 42,837,025 determinants when using a ghost atom rather than 2,123,544 with symmetry and no ghost atom.

**Figure 12:**
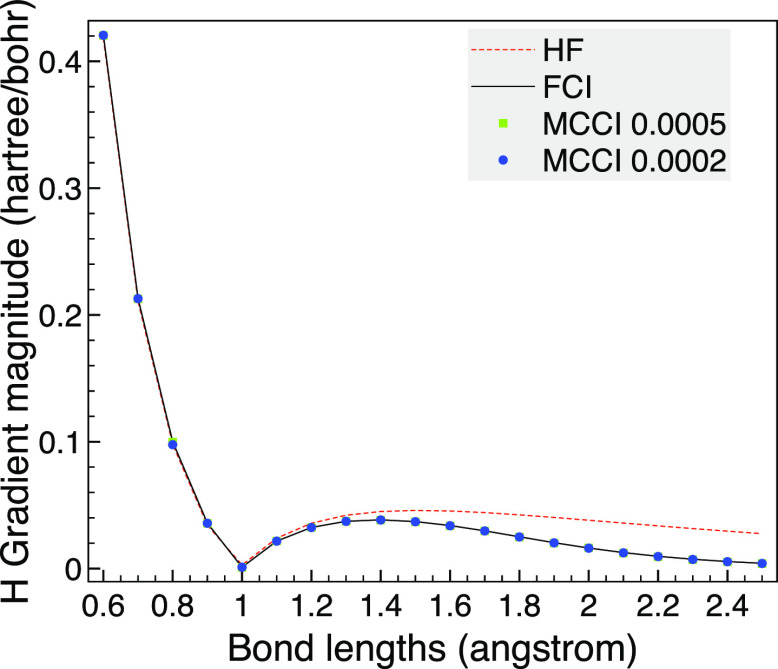
Magnitude of the gradient vector of a hydrogen atom in hexagonal planar H_6_ against bond lengths using the cc-pVDZ basis set and the D_2h_ point group.

We now vary the bond length of hexagonal planar H_6_ when using D_2h_ to appraise the accuracy of MCCI for its analytic gradients. We see in Figure 12 that the magnitude of the gradient vector is essentially FCI quality on the scale of the graph when using MCCI with just a slight difference between MCCI results with different cutoffs at a bond length of 0.8 Å. In contrast HF is noticeably different to FCI as the bonds are stretched toward dissociation.

We quantify the gradient accuracy compared with FCI in Table 5, where we see that the MCCI results reduce the error of the HF calculation by more than an order of magnitude, and the MCCI wave functions use fewer than ten thousand determinants in contrast to the FCI space of around two million determinants. There is a slight increase in the gradient error on lowering the MCCI cutoff from 5 × 10^–4^ to 2 × 10^–4^, highlighting again the nonvariational nature and challenge of the gradient, but the errors for both cutoffs are very small.

**Table 5:** Errors Using RMSE When Compared with FCI for the Gradient of Ground-State Hexagonal Planar H_6_ and Mean Number of Determinants across the 20 Bond Lengths Considered When Employing the cc-pVDZ Basis

Method	Gradient Error (hartree/bohr)	Mean Determinants
HF	0.868 × 10^–2^	1
MCCI 1 × 10^–3^	0.0353 × 10^–2^	1305
MCCI 5 × 10^–4^	0.0180 × 10^–2^	2527
MCCI 2 × 10^–4^	0.0217 × 10^–2^	7131

### 3.5 Methane

Finally we consider CH_4_ as with tetrahedral geometry its full point group is *T*_*d*_, which has irreps of dimension 3, so triply degenerate orbitals are possible by symmetry. With the cc-pVDZ basis, we find eight sets of triply degenerate orbitals and two sets of doubly degenerate orbitals, but each set splits into different irreps when the *C*_2*v*_ subgroup is used. Starting from the tetrahedral geometry with bond lengths of 1 bohr, we slightly break the symmetries by shifting the x coordinate of one hydrogen by 0.01 bohr and the y position of another by −0.01 bohr with the aim of bringing about near degeneracies. With the MCCI wave function using a cutoff of 5 × 10^–4^ for the *A*_1_ ground state, all pairs of orbitals have non-negligible *ΔF*. We find that the smallest gap between orbital energies that need to be considered is now 2.3 × 10^–5^ hartree.

**Figure 13:**
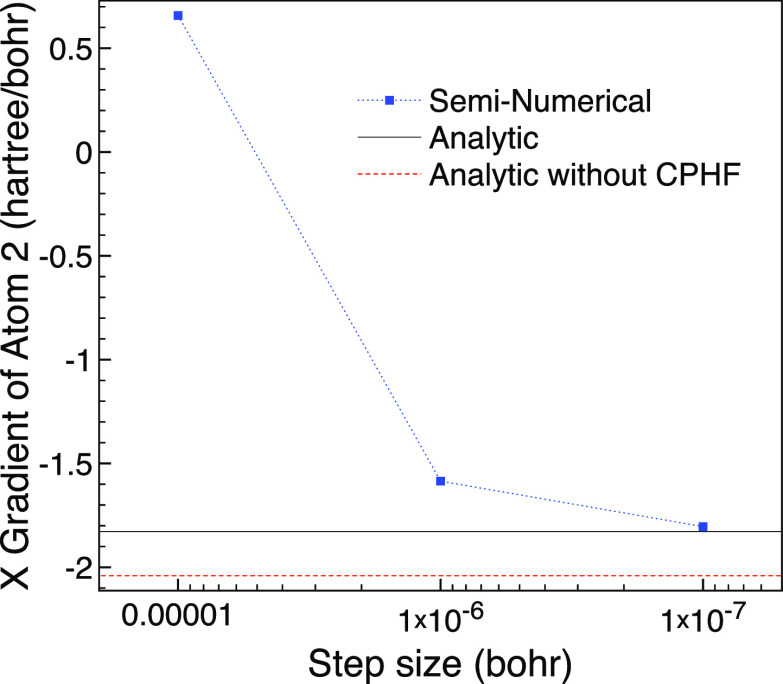
Comparison of seminumerical and analytic X gradient of a hydrogen atom in broken symmetry CH_4_ based on a tetrahedral geometry with 1 bohr bond lengths using the cc-pVDZ basis set and one frozen orbital with MCCI using a cutoff of 5 × 10^–4^.

We see in Figure 13 that the seminumerical MCCI result for the X gradient of a hydrogen approaches the analytic result as the step size is lowered. This system appears to be the most challenging for the seminumerical gradient as a step size of 10^–5^ bohr has the wrong sign. For NH_3_ with a smaller basis we saw that this step size could give qualitatively correct gradients for both of the broken symmetry test cases (Figures 4 and 5). However, this CH_4_ example, in addition to a larger basis set and more near-degenerate orbitals, also has short bond lengths so the gradient is relatively large and changing rapidly.

**Figure 14:**
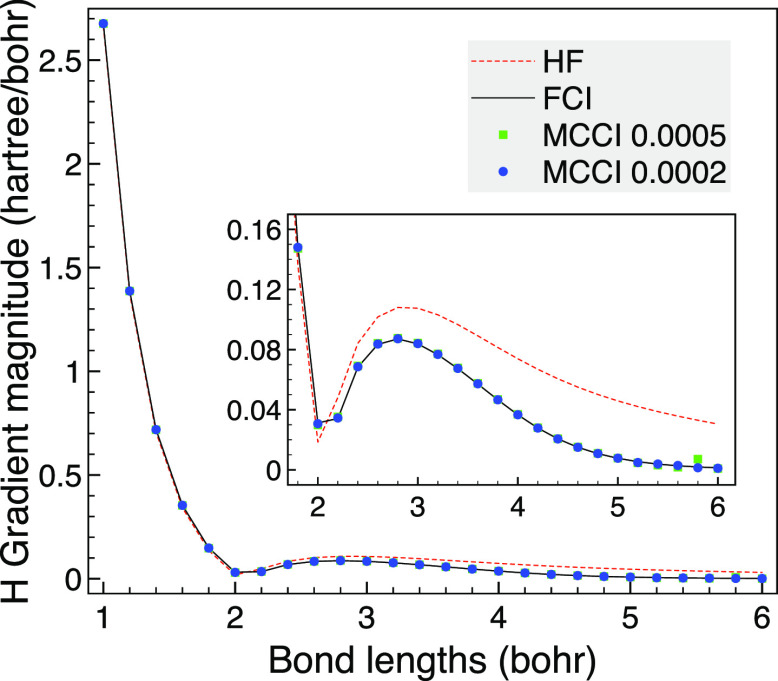
Magnitude of the gradient vector for a hydrogen atom in CH_4_ against bond lengths for the tetrahedral geometry using the 6-31G basis set with one frozen orbital and the *C*_2*v*_ point group. Inset: Enlarged view of the gradient curve.

We now use the tetrahedral geometry and a smaller basis so that we can more easily calculate the FCI analytic gradients using MCSCF in MOLPRO^58^ for comparison. We use the 6-31G basis with one frozen orbital and plot the magnitude of the analytic gradient for a hydrogen atom as the bond length is varied in Figure 14. We see that the MCCI analytic gradient magnitudes are essentially indistinguishable from FCI on the scale of the graph when a cutoff of 2 × 10^–4^ is used. There is only a slight discrepancy when a larger cutoff of 5 × 10^–4^ is used and the bonds are stretched to 5.8 bohr (inset of Figure 14) which may be due to the use of Slater determinants allowing a different spin state to be converged on. The inset also shows how at longer bond lengths the HF gradient magnitude is noticeably different to FCI and MCCI.

The accuracy is quantified in Table 6 where we see that the error can be reduced by an order of magnitude through using MCCI with a cutoff of 10^–3^ compared with HF. The accuracy can be improved by around another order of magnitude by lowering the MCCI cutoff to 2 × 10^–4^. This is similar to the general behavior for the other molecules considered in this work and again this was achieved using a very small fraction of the FCI space which comprises 828,944 determinants for this system.

**Table 6:** Errors Using RMSE When Compared with FCI for the Gradient of the Ground *A*_1_ State of Tetrahedral CH_4_ and the Mean Number of Determinants across the 26 Bond Lengths Considered When Employing 6-31G and One Frozen Orbital

Method	Gradient Error (hartree/bohr)	Mean Determinants
HF	1.48 × 10^–2^	1
MCCI 1 × 10^–3^	0.133 × 10^–2^	2560
MCCI 5 × 10^–4^	0.23 × 10^–2^	5509
MCCI 2 × 10^–4^	0.0202 × 10^–2^	14692

## 4 Conclusions

We have developed efficient analytic gradients for selected configuration interaction (CI) wave functions and demonstrated them on carbon monoxide, ammonia, square planar H_4_, hexagonal planar H_6_, and methane. The selected CI approach of Monte Carlo configuration interaction (MCCI) was shown to be able to produce full configuration interaction (FCI) quality gradients for these systems despite using a very small fraction of the FCI determinants.

In contrast to analytic gradients for a standard configuration interaction truncated to singles and doubles (CISD), for example, the selected CI energy is not necessarily invariant to rotations between orbitals that are occupied in the Hartree–Fock wave function (or unoccupied) and all pairs of orbitals might have to be considered for the derivative terms connected to a change in the molecular orbital coefficients. These are calculated using the coupled perturbed Hartree–Fock (CPHF) equations that can, in principle, have problems if pairs of unoccupied orbitals have to be considered and they have (near) degeneracies. We proved that orbital pairs belonging to different irreducible representations did not need to have their CPHF contribution calculated, and found that degenerate orbitals split into different irreducible representations when the largest abelian subgroup was used. We tested the analytic approach on near degeneracies by slightly changing the geometry of trigonal planar ammonia, which originally has doubly degenerate orbitals, square planar H_4_, hexagonal planar H_6_, which originally has doubly degenerate orbitals, and tetrahedral methane, which originally has triply degenerate orbitals, to break symmetry. We found that, at least for these systems, near degeneracies did not cause a problem for the analytic selected CI derivatives by verifying the results with the introduced seminumerical approach. This was despite the change in geometry that removed symmetries being as small as 10^–3^ Å for ammonia. However, the seminumerical derivatives tended to require smaller step sizes for accurate results as the systems became more challenging. This suggests that the selected CI analytic gradients will be able to cope with getting very close to a high-symmetry structure, if necessary, when starting from one without symmetry in geometry optimization or dynamics.

When varying bond lengths for the molecules we found that the plotted analytic gradients were essentially indistinguishable from FCI when using MCCI with a cutoff of 2 × 10^–4^ except for one geometry of the challenging square planar H_4_ where MCCI had difficulties finding a multireference wave function rather than an essentially single reference one. The accuracy was quantified using the root-mean-square error. We found that, for these molecules, MCCI with a cutoff of 1 × 10^–3^ gave around an order of magnitude improvement on Hartree–Fock, and for ammonia and methane, this was enhanced by around another order of magnitude on lowering the cutoff to 2 × 10^–4^ while only a very small fraction of the FCI space was used.

This proof of concept for analytic selected CI gradients was demonstrated to work well using Monte Carlo configuration interaction with Slater determinants. However, we emphasize that it can be used for general selected CI methods and would be expected to be further improved by, for example, using different methods of selection, configuration state functions (CSFs), and the wave function from a previous geometry as a starting point.

These selected CI analytic gradients enable higher accuracy than seminumerical derivatives without the dependence on step size, and better efficiency by reducing the computational scaling by around the number of degrees of freedom. Hence, future work can use these for efficient geometry optimization of multireference problems and build on the selected CI analytic gradient machinery for selected CI analytic nonadiabatic couplings for dynamics.
